# The effectiveness of acupoint application in the treatment of dysphagia after stroke: a systematic review and meta-analysis

**DOI:** 10.3389/fneur.2026.1770892

**Published:** 2026-03-25

**Authors:** Xianxian Qian, Jinrui Cui, Yujing Xu, Tanfang Wei, Lin Yang, Zhongxia Fu

**Affiliations:** 1 School of Nursing, Gansu University of Chinese Medicine, Lanzhou, China; 2 Department of Neurology, Gansu Provincial Maternity and Child Care Hospital (Gansu Provincial Central Hospital), Lanzhou, China

**Keywords:** acupoint application, dysphagia, stroke, meta-analysis, systematic review

## Abstract

**Introduction:**

The systematic review aimed to evaluate the efficacy of acupoint application for patients with post-stroke dysphagia,to inform its clinical adoption in rehabilitation.

**Methods:**

We searched PubMed, the Cochrane Library, Embase, Web of Science, CNKI, CBM, Wanfang, and VIP from their inception to February 24, 2025, for randomized controlled trials (RCTs) on acupoint application for the treatment of post-stroke dysphagia. Two researchers independently extracted information according to inclusion and exclusion criteria. Data analysis was performed using RevMan 5.4.1 software.

**Results:**

The study comprised 13 RCTs involving 1,344 people. The results indicated that acupoint application combined with control treatment was superior to control treatment alone in improving the effective rate [RR = 3.52, 95% CI (2.41, 5.15), *P* < 0.00001], Videofluoroscopic Swallowing Study (VFSS) scores [MD = 1.86, 95% CI (1.59, 2.14), *P* < 0.00001], Swallowing Quality of Life (SWAL-QOL) scores [MD = 24.98, 95% CI (18.82, 31.14), *P* < 0.00001], and serum albumin levels [MD = 4.35, 95% CI (2.50, 6.20), *P* < 0.00001]. Furthermore, the intervention reduced scores on the water swallow test [MD = −1.38, 95% CI (−1.95, −0.82), *P* < 0.00001] and the Nutritional Risk Screening 2002 (NRS2002) [MD = −0.54, 95% CI (−1.05, −0.04), *P* = 0.03].

**Discussion:**

The acupoint application may be a potentially beneficial complementary therapy for dysphagia after stroke. However, the current evidence is of low certainty. More high-quality evidence is needed to confirm the effcacy of acupoint application in treating post-stroke dysphagia.

**Systematic review registration:**

https://www.crd.york.ac.uk/prospero/display_record.php?ID=CRD420251011807, PROSPERO identifier: CRD420251011807.

## 1 Introduction

Stroke is an acute cerebrovascular disease caused by various etiologies that lead to an impaired blood supply to the brain, resulting in brain tissue damage (1). It is a major cause of disability and death worldwide (2). Approximately 50%−67% of stroke patients experience dysphagia, which can lead to malnutrition, dehydration, and aspiration pneumonia, significantly impacting patient prognosis and quality of life (3–6). As a common external treatment method in traditional Chinese medicine, acupoint application facilitates the transdermal absorption of medicinal substances and stimulates relevant meridians and acupoints. It has the function of regulating the qi and blood of internal organs and meridians, which can improve the therapeutic effect of swallowing disorders.

In recent years, clinical studies on acupoint application for post-stroke dysphagia have gradually increased. However, the current evidence regarding the efficacy of acupoint application for patients with post-stroke dysphagia remains inconsistent. Therefore, this study aims to systematically review the existing literature to evaluate the effectiveness of acupoint application in patients with post-stroke dysphagia, providing a certain reference for clinical treatment.

## 2 Materials and methods

### 2.1 Study registration

According to the guidelines (7), this systematic review protocol is registered in PROSPERO (No. CRD420251011807).

### 2.2 Inclusion criteria

Population: Patients diagnosed with dysphagia after stroke, where the diagnostic criteria for stroke refer to the “Guidelines for Prevention and Treatment of Cerebrovascular Diseases” (8), and confirmed dysphagia via recognized diagnostic methods. Intervention: The experimental group was treated with acupoint application therapy on the basis of the control group. Control: The control group received conventional rehabilitation training or was combined with other therapies (such as acupuncture or neuromuscular electrical stimulation). Outcome: the effective rate; water swallow test (WST) (9), Videofluoroscopic Swallowing Study (VFSS) score (10), Swallowing Quality of Life questionnaire (SWAL-QOL) score (11), serum albumin (ALB) levels, and Nutritional Risk Screening 2002 (NRS2002) score. Study type: Only randomized controlled trials (RCTs) were included.

### 2.3 Exclusion criteria

(1) Combined with other diseases; (2) Repeatedly published literature; (3) Studies with incomplete data; (4) Conference proceedings; (5) Master's and doctoral dissertations.

### 2.4 Search strategy

The literature search was performed across eight databases: PubMed, the Cochrane Library, Embase, Web of Science, China Knowledge Infrastructure (CNKI), China Science Journal Database (VIP), Wan-fang Database, and China Biomedical Literature Service System (CBM). All from the inception to February 24, 2025. The search terms include “stroke,” “apoplexy,” “brain infarct,” “cerebral infarction,” “cerebral embolism,” “cerebral hemorrhage,” “cerebral ischemia,” “cerebral vascular accident,” “cerebrovascular accident,” “cerebrovascular disorder,” “deglutition disorder,” “dysphagia,” “swallowing disorder,” “difficulty in swallowing,” “acupoint application,” and “acupoint sticking.” Using PubMed search strategy as an example, refer to Table 1.

**Table 1:** PubMed session results.

**Search**	**Query**
#1	“Stroke” [MeSH Terms] OR “stroke”[Title/Abstract] OR “apoplexy”[Title/Abstract] OR “brain infraction”[Title/Abstract] OR “cerebral infarction”[Title/Abstract] OR “cerebral ischemia”[Title/Abstract] OR “cerebral embolism”[Title/Abstract] OR “cerebral hemorrhage”[Title/Abstract] OR “cerebrovascular accident^*^”[Title/Abstract] OR “brain vascular accident^*^”[Title/Abstract] OR “cerebrovascular disorder”[Title/Abstract]
#2	“Dysphagia” [MeSH Terms] OR “dysphagia”[Title/Abstract] OR “swallowing disorder^*^”[Title/Abstract] OR “deglutition disorder^*^”[Title/Abstract] OR “difficulty in swallowing”[Title/Abstract]
#3	“acupoint application” [MeSH Terms] OR “acupoint sticking”[Title/Abstract]
#4	#1 AND #2 AND #3

### 2.5 Literature screening and data extraction

Researchers used EndNote 20 to exclude duplicate documents, with two investigators independently screening the literature according to inclusion and exclusion criteria, ultimately identifying studies that met the predetermined inclusion standards. Subsequently, the relevant information was independently extracted from the included studies by two researchers. Including the first author, sample size, age, disease duration, intervention measures, treatment frequency, treatment duration, outcome indicators, and so on. Any disagreements encountered during the literature screening and data extraction process should be resolved first through discussion between the two investigators, and if consensus cannot be reached, the matter should be adjudicated by a third reviewer.

### 2.6 Risk bias and quality assessment

The methodological quality of all included studies was assessed using the Cochrane Risk of Bias Tool. Each study was independently reviewed by two investigators across seven domains: random sequence generation, allocation concealment, blinding of participants and outcome assessors, selective reporting, incomplete outcome data, and other sources of bias, with the risk of bias categorized as “low,” “high,” or “unclear.” Disagreements during assessment were addressed through discussion with a third independent reviewer.

### 2.7 Statistical analysis

The meta-analysis was conducted with *RevMan 5.4.1* software, employing the odds ratio (OR) and mean difference (MD) for dichotomous and continuous variables, both expressed with 95% confidence intervals (CI). For heterogeneity testing, if *P* > 0.1 and *I*^2^ < 50%, a fixed-effect model was used; otherwise, a random-effects model was applied. If necessary, subgroup analysis and sensitivity analysis are performed. When the number of included studies exceeded 10, a funnel plot was constructed.

## 3 Results

### 3.1 Literature search results

**Figure 1:**
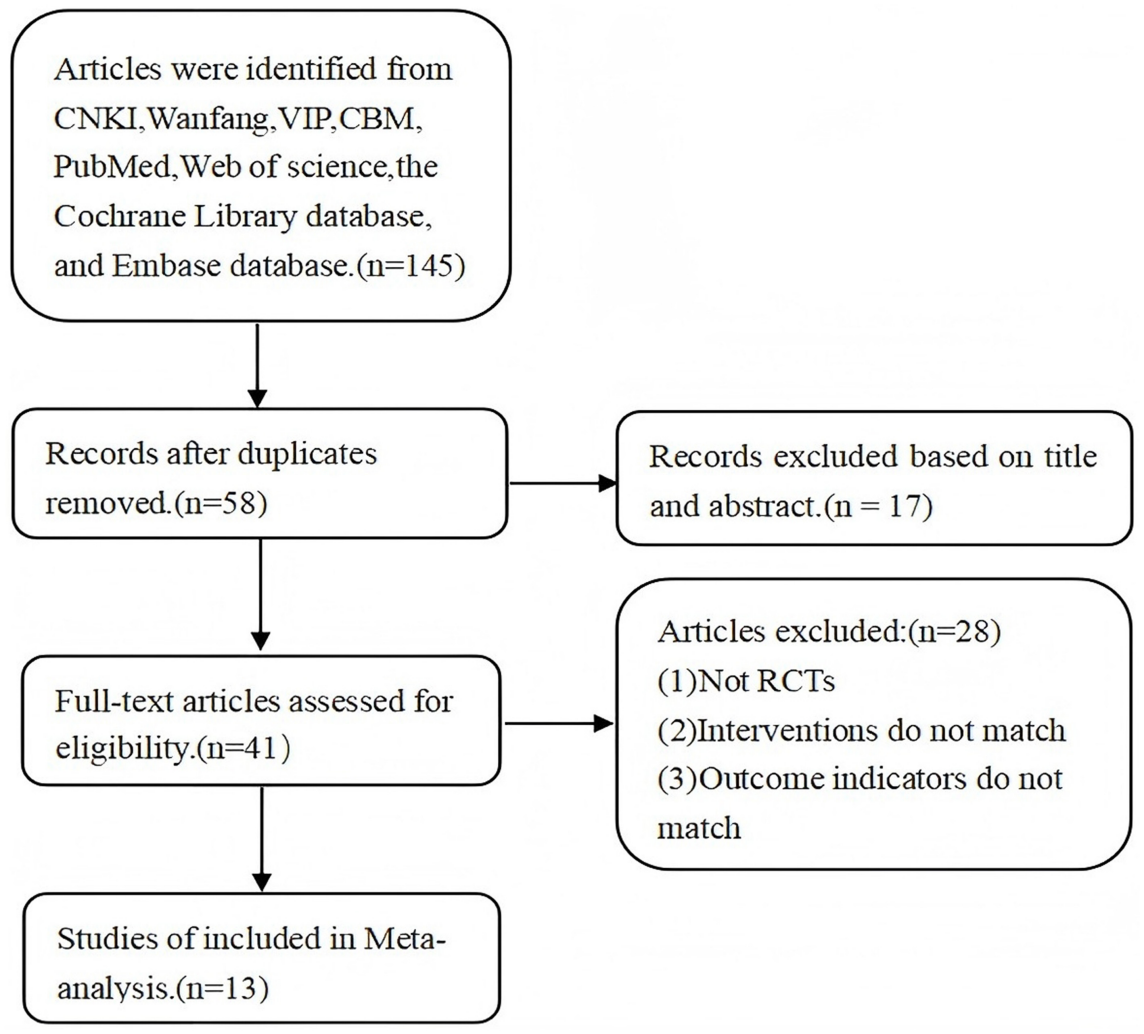
Literature screening process.

A total of 145 relevant studies were retrieved. After screening based on inclusion and exclusion criteria, 13 RCTs were ultimately included. The literature screening flowchart is shown in Figure 1.

### 3.2 Basic characteristics of the studies

A total of 13 studies were included (12–24), involving 1,344 participants, with 672 in the experimental group and 672 in the control group. The publication period is between 2013 and 2024. Among these 13 studies, 11 studies (12–20, 22, 23) reported the effective rate, 6 studies (14, 15, 19, 20, 23, 24) reported VFSS scores, 5 studies (16, 19–21, 24) reported the water swallow test, 5 studies (15, 19, 21–23) reported SWAL-QOL scores, 4 studies (15, 17, 18, 22) reported serum albumin content, and 3 studies (15, 18, 22) reported NRS2002 scores. Among all the included studies, the experimental group received acupoint application combined with control treatment. In the control group, ten studies adopted conventional rehabilitation training, two studies used conventional rehabilitation training plus acupuncture, and one study employed conventional rehabilitation training plus neuromuscular electrical stimulation (NMES), as detailed in Table 2.

**Table 2:** Characteristics of 13 studies.

**Reference**	**Sample size (E/C)**	**Age (E/C)**	**Gender (M/F)**	**Disease duration**	**Intervention**		**Acupoint application**		**Outcome**
					**Methods**	**Duration**	**Frequency**	**Acupoints**	
Zhao et al. (12)	23 (15/8)/23 (13/10)	60.10 ± 8.42/56.27 ± 10.36	E:15/8 C:13/10	NA	E:FT+AA C:FT	1 m	6 h/d, 6 d/w	TianTu (CV22), LianQuan (RN23), RenYing (ST9)	①
Lai (13)	50 (30/20)/50 (31/19)	64 ± 6/ 63 ± 6	E:30/20 C:31/19	NA	E:FT+AA C:FT	1 m	0.5 h/d, 7 d/w	FengChi (GB20), HeGu (LI4), JinJin (EX-HN12)	①
Luo et al. (14)	35 (18/17)/35 (16/19)	59.60 ± 3.82/ 60.23 ± 3.97	E:18/17 C:16/19	32.81 ± 11.02 h 29.30 ± 10.80 h	E:FT+AA C:FT	2 w	0.5 h/d, 7 d/w	LianQuan (RN23)	①③
Hu (15)	61 (37/24)/61 (35/26)	65.3 ± 12.2/65.3 ± 12.2	E:37/24 C:35/26	19.4 ± 5.7 d 19.2 ± 5.6 d	E:FT+AA C:FT	1 m	6 h/d, 6 d/w	TianTu (CV22), LianQuan (RN23), RenYing (ST9)	①③④⑤⑥
Zheng et al. (16)	42 (23/19)/42 (24/18)	62.6 ± 7.5/63.4 ± 7.9	E:23/19 C:24/18	7.0 ± 6.4 d 6.0 ± 7.6 d	E:FT+NMES+AA C:FT+NMES	2 w	4–6 h/d, 7 d/w	TianTu (CV22), LianQuan (RN23), RenYing (ST9), FuTu(LI18)	①②
Dong et al. (17)	46 (27/19)/46 (26/20)	65.51 ± 5.72/65.49 ± 5.66	E:27/19 C:26/20	19.21 ± 4.18 d 19.15 ± 4.25 d	E:FT+AA C:FT	4 w	4 h/d, 6 d/w	TianTu (CV22), LianQuan (RN23), RenYing (ST9)	①⑤
Wu et al. (18)	40 (22/18)/ 40 (21/19)	67.34 ± 7.41/67.27 ± 7.32	E:22/18 C:21/19	3.73 ± 0.76 m 3.65 ± 0.89 m	E:FT+AA C:FT	1 m	6 h/d, 7 d/w	TianTu (CV22), LianQuan (RN23), RenYing (ST9)	①⑤⑥
Ma et al. (19)	60 (26/29)/60 (24/31)	63.5 ± 11.4/64.3 ± 11.2	E:26/29 C:24/31	25.4 ± 7.8 d 25.2 ± 7.6 d	E:FT+AA C:FT	20 d	6 h/d, 6 d/w	LianQuan (RN23), Bilateral GongXue Bilateral RenYing (ST9)	①②③④
Zhou et al. (20)	75 (45/30)/75 (46/29)	52.03 ± 7.06/51.97 ± 6.66	E:45/30 C:46/29	1–8 d 1–7 d	E:FT+A+AA C:FT+A	3 w	4–6 h/d, 7 d/w	FengFu (GV16), LianQuan (RN23), RenYing (ST9)	①②③
Jiao (21)	40 (25/15)/ 40 (24/16)	63.15 ± 5.09/63.06 ± 5.17	E:25/15 C:24/16	NA	E:FT+AA C:FT	4 w	6 h/d, 6 d/w	TianTu (CV22), LianQuan (RN23), RenYing (ST9)	②④
Wang (22)	35 (21/14)/35 (23/12)	65.24 ± 5.39/65.18 ± 5.45	E:21/14 C:23/12	3.11 ± 0.43 m 3.09 ± 0.56 m	E:FT+AA C:FT	1 m	6 h/d, 6 d/w	TianTu (CV22), LianQuan (RN23), RenYing (ST9)	①④⑤⑥
Yu and Wu (23)	75 (42/33)/75 (40/35)	62.92 ± 10.56/63.45 ± 11.04	E:42/33 C:40/35	21.56 ± 5.80 d 22.18 ± 5.25 d	E:FT+AA C:FT	4 w	6 h/d, 6 d/w	TianTu (CV22), LianQuan (RN23), RenYing (ST9)	①③④
Zhang et al. (24)	90 (50/40)/90 (49/41)	56.44 ± 7.88/ 56.77 ± 7.83	E:50/40 C:49/41	6.09 ± 0.85 d 5.88 ± 0.72 d	E:FT+A+AA C:FT+A	3 w	6 h/d, 6 d/w	LianQuan (RN23), Bilateral GongXue, Bilateral RenYing (ST9)	②③

E, experimental group; C, control group; F, male; M, male; h, hour; d, day; w, week; m, month; RT, Rehabilitation training; AA, acupoint application; NMES, Neuromuscular electrical stimulation; A, acupuncture; ①, The effective rate; ②, Water swallow test; ③, VFSS scores; ④, SWAL-QOL scores; ⑤, ALB; ⑥, NRS2002 scores.

### 3.3 Methodological and reporting quality

**Figure 2:**
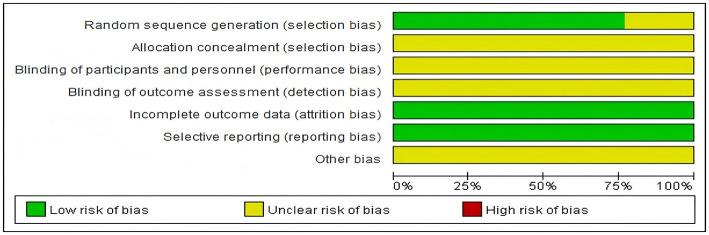
Risk of bias graph.

**Figure 3:**
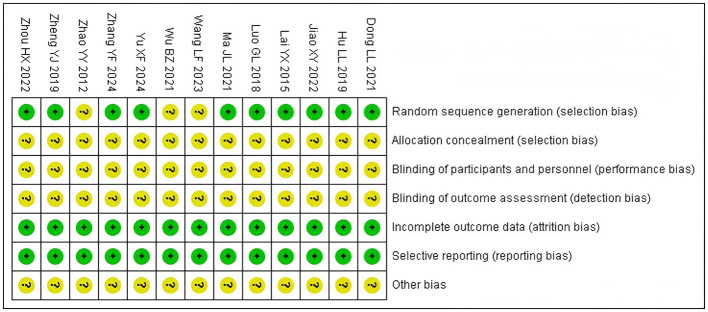
Risk of bias summary.

Among the 13 included studies, 3 studies (12, 18, 22) only mentioned randomization, while the remaining (13–17, 19–21, 23, 24) clearly specified random allocation methods. None of the included studies mentioned allocation concealment or blinding. No missing data or selective reporting was identified. Other potential biases were not described, and the risk of bias was unclear. Methodological quality assessment of included studies. See Figures 2, 3.

### 3.4 Results of the meta-analysis

#### 3.4.1 The effective rate

**Figure 4:**
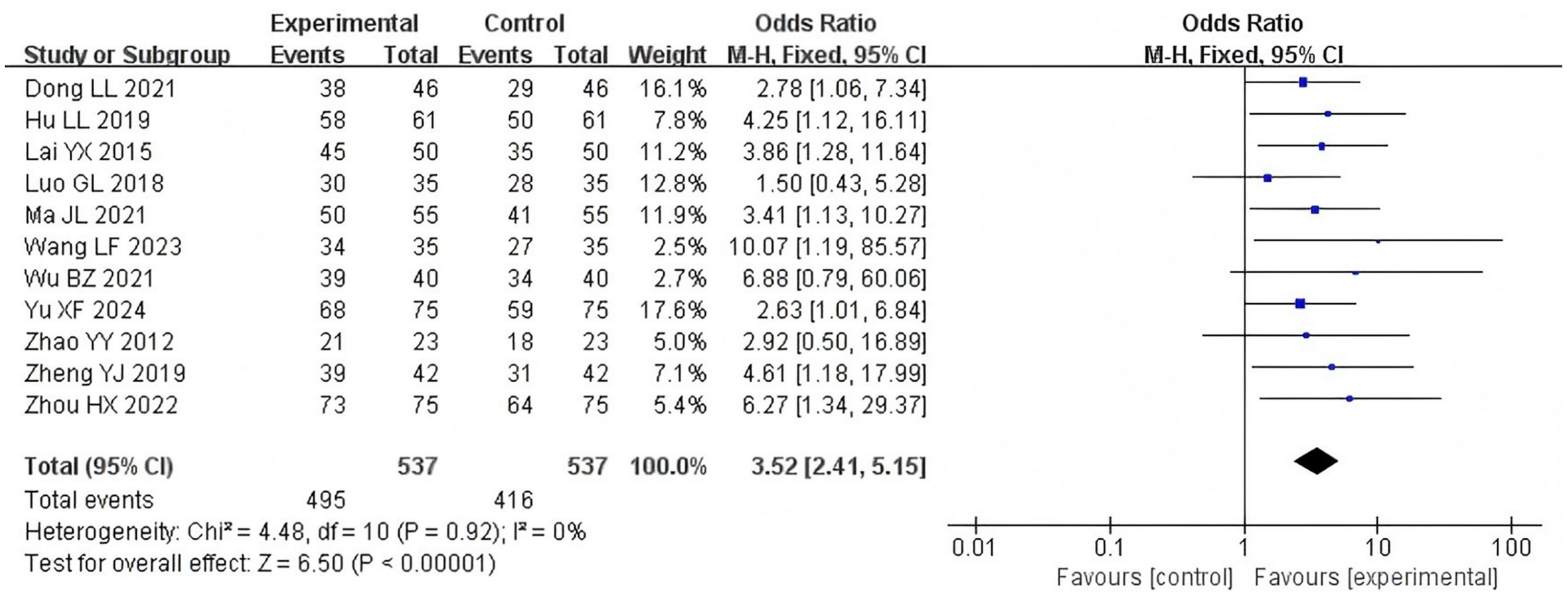
Forest plot of effective rate comparisons between groups.

Eleven studies (12–20, 22, 23) reported treatment efficacy rates, involving 1,074 patients. There was no significant heterogeneity between the three RCTs (*P* = 0.92, *I*^2^ = 0%), a fixed-effect model was applied. The results suggested that swallowing function was better in the acupoint application treated group compared with the control group without acupoint application [RR = 3.52, 95% CI (2.41, 5.15), *Z* = 6.50, *P* < 0.00001, Figure 4].

#### 3.4.2 VFSS scores

**Figure 5:**
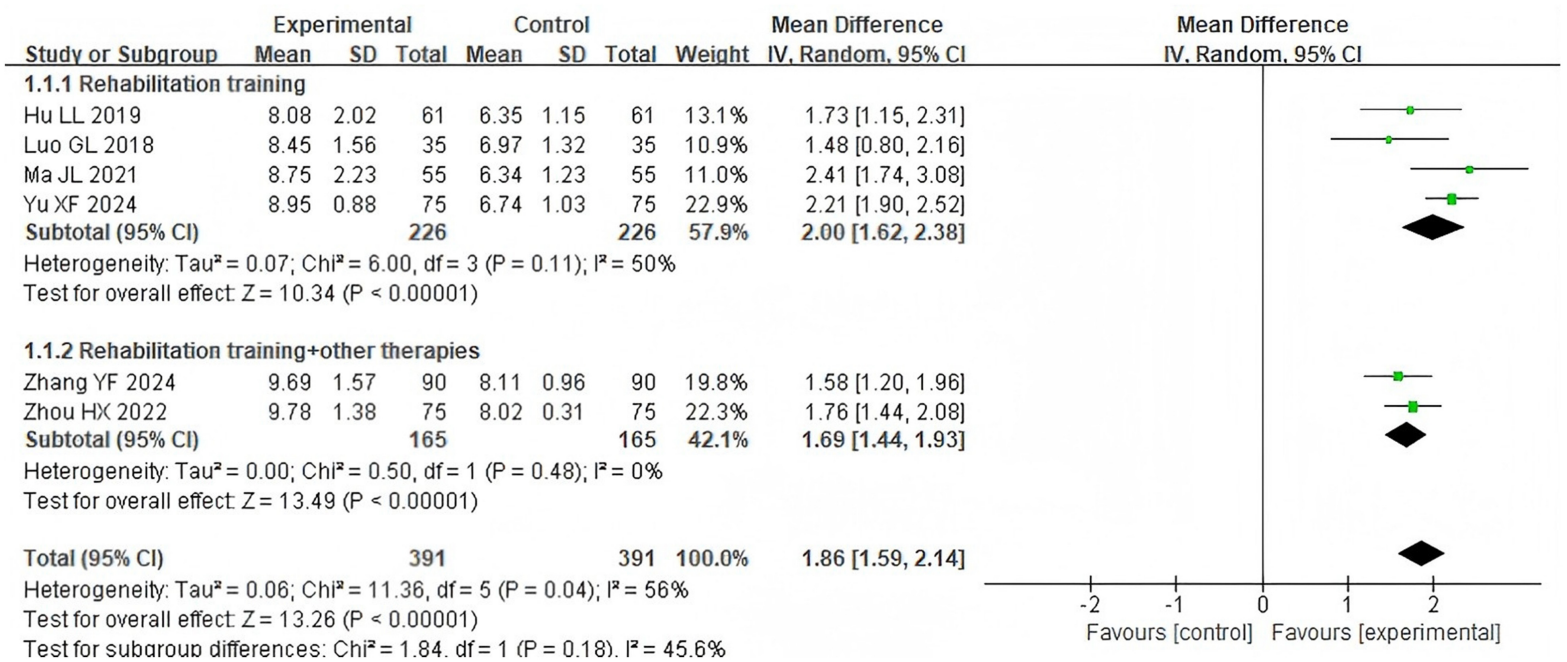
Forest plot of VFSS scores subgroup analyses stratified by control types.

Six studies (14, 15, 19, 20, 23, 24) reported the VFSS scores, involving 782 patients. Due to moderate heterogeneity among the studies (*P* = 0.04, I^2^ = 56%), a random-effects model was applied. The results demonstrated that the VFSS score was improved in the acupoint application group compared with the control group [MD = 1.86, 95% CI (1.59, 2.14), Z = 13.26, *P* < 0.00001]. Subgroup analysis by control group intervention methods revealed that compared with conventional rehabilitation training alone [MD = 2.00, 95% CI (1.62, 2.38), Z = 10.34, *P* < 0.00001] or conventional rehabilitation training combined with other therapies [MD = 1.69, 95% CI (1.44, 1.93), Z = 13.49, *P* < 0.00001], the acupoint application group could lead to higher VFSS scores. However, the variation across subgroups was not statistically significant (*P* = 0.18, Figure 5). Subgroup analysis based on intervention duration revealed no statistically differences among subgroups (*P* = 0.32).

#### 3.4.3 Water swallow test

**Figure 6:**
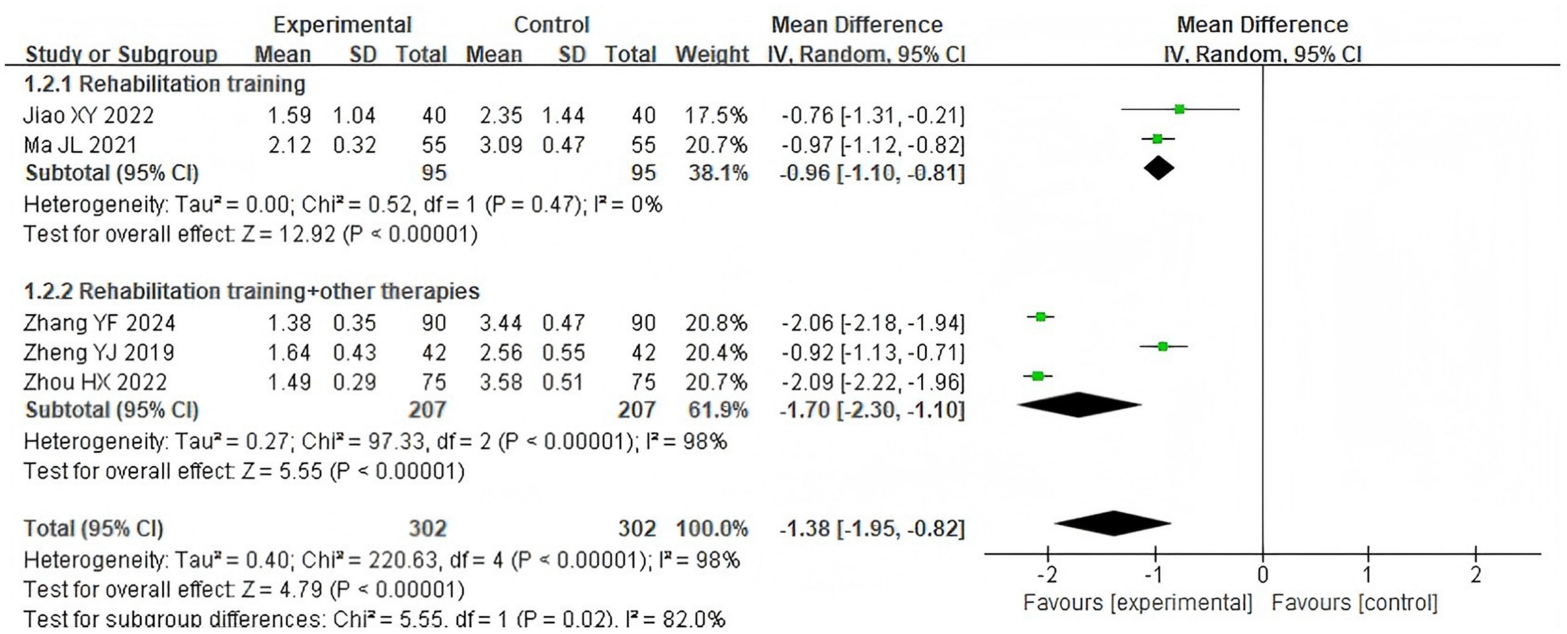
Forest plot of water swallow test subgroup analyses stratified by control types.

Five studies (16, 19–21, 24) reported the results of the water swallow test, involving 604 patients. Due to high heterogeneity between studies (*P* < 0.00001, *I*^2^ = 98%), a random-effects model was applied. The results showed that the acupoint application group had significantly reduced water swallow test scores compared with the control group [MD = −1.38, 95% CI (−1.95, −0.82), *Z* = 4.79, *P* < 0.00001]. Subgroup analysis based on the control group intervention method indicated that, compared with conventional rehabilitation training alone [MD = −0.96, 95% CI (−1.10, −0.81), Z = 12.92, *P* < 0.00001] or conventional rehabilitation combined with other therapies [MD = −1.70, 95% CI (−2.30, −1.10), Z = 5.55, *P* < 0.00001], the acupoint application group could further reduce the water swallowing test scores as detailed in Figure 6. The high heterogeneity may be attributed to differences in intervention methods across the control groups. Since only one intervention lasted more than 3 weeks among the five studies, subgroup analysis of this metric was not performed based on intervention duration.

#### 3.4.4 SWAL-QOL scores

**Figure 7:**
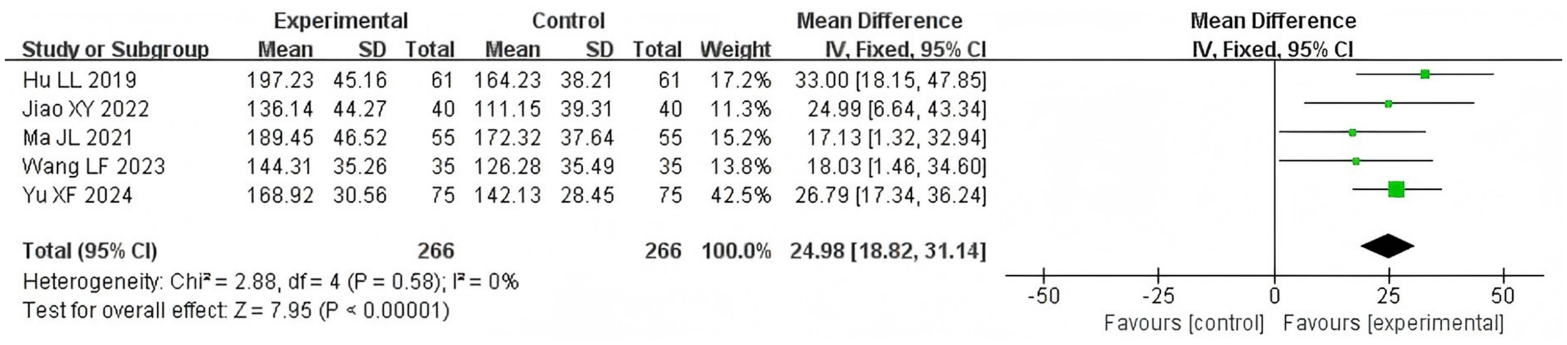
Forest plot of SWAL-QOL score comparisons between groups.

A total of 532 patients in 5 studies (15, 19, 21–23) reported SWAL-QOL scores for both groups. There was no significant heterogeneity between studies (*P* = 0.58, I^2^ = 0%), a fixed-effects model was applied. The results show that compared to the control group, the acupoint application group could improve the SWAL-QOL scores [MD = 24.98, 95% CI (18.82, 31.14), *Z* = 7.95, *P* < 0.00001, Figure 7].

#### 3.4.5 ALB

**Figure 8:**
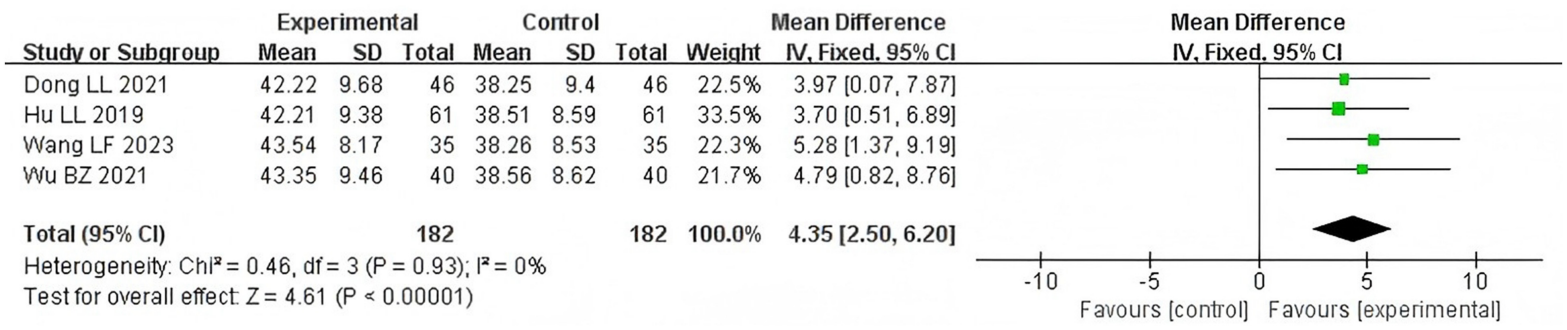
Forest plot of ALB level comparisons between groups.

Four studies (15, 17, 18, 22) involving 364 patients reported serum albumin levels in two groups. There was no significant heterogeneity between studies (*P* = 0.93, *I*^2^ = 0%), a fixed-effects model was applied. Results indicate that the acupoint application group significantly enhances serum albumin levels compared to the control group [MD = 4.35, 95% CI (2.50, 6.20), *Z* = 4.61, *P* < 0.00001]. See Figure 8.

#### 3.4.6 NRS2002 scores

**Figure 9:**
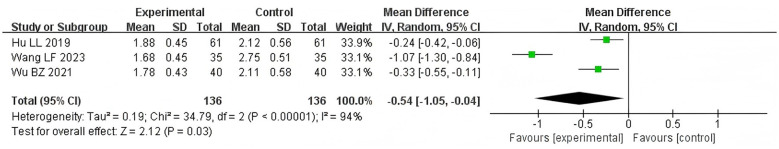
Forest plot of NRS2002 scores comparisons between groups.

Three studies (15, 18, 22) involving 272 patients reported NRS2002 scores for both groups. Due to high heterogeneity between studies (*P* < 0.00001, *I*^2^ = 94%), a random-effects model was applied. Results indicate that compared to the control group, the acupoint application group could reduce NRS2002 scores [MD = −0.54, 95% CI (−1.05, −0.04), *Z* = 2.12, *P* = 0.03]. See Figure 9. No subgroup analysis was carried out, as the number of included studies was limited.

#### 3.4.7 Security evaluation

Adverse reactions were reported in only one out of the 13 included studies. The intervention group had one case of aspiration, whereas the control group had six, indicating a lower incidence of aspiration in the intervention group during treatment. However, the clinical safety of acupoint application for post-stroke dysphagia requires further investigation.

### 3.5 Sensitivity analysis

Sensitivity analyses were conducted on the efficacy rate, water swallow test, VFSS scores, SWAL-QOL scores, serum albumin levels, and NRS2002 scores, using a method of sequentially excluding each study. The results demonstrated that after removing any single study and re-conducting the meta-analysis, the pooled effect sizes remained stable in overall direction and statistical significance. These findings confirm that the results of this meta-analysis have good stability and reliability.

### 3.6 Risk of publication bias assessment

**Figure 10:**
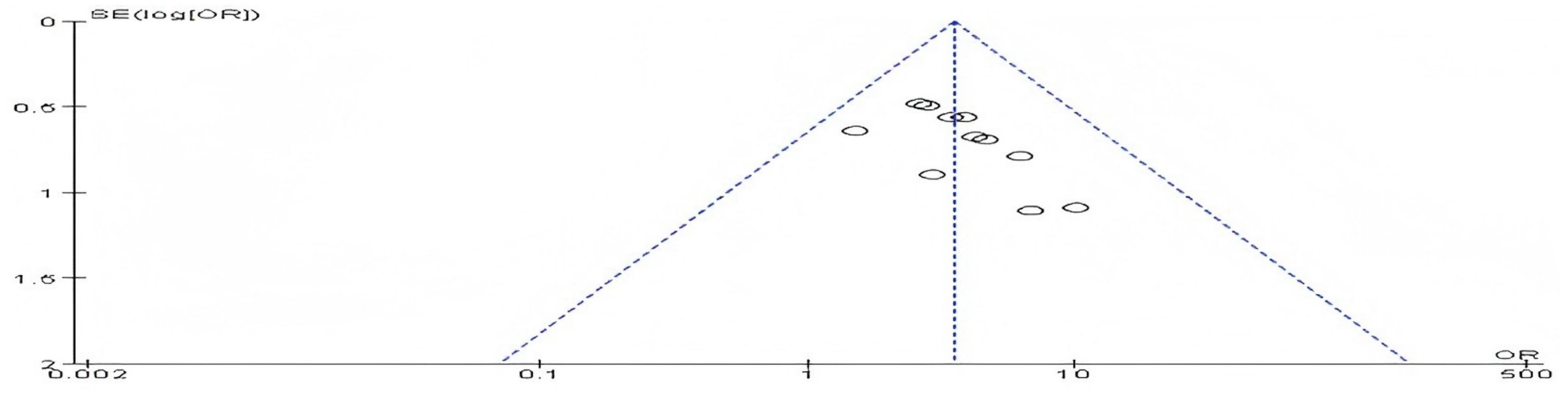
Funnel plot of effective rate.

Eleven studies reported the efficacy rate. The generated funnel plot is not completely symmetrical, indicating that there may be publication bias in this research. See Figure 10.

## 4 Discussion

The objective of this meta-analysis was to investigate the effectiveness of acupoint application for patients with post-stroke dysphagia. After merging data from 13 RCTs, the results show that acupoint application combined with conventional rehabilitation training was superior to the conventional rehabilitation training alone in improving the treatment effective rate, water swallow test scores, VFSS scores, SWAL-QOL scores, serum albumin levels, and NRS2002 score in patients with dysphagia after stroke. However, these findings should be interpreted with caution, as the included studies carry a high risk of bias (lack of allocation concealment and blinding) and small sample sizes, which may lead to overestimation of the treatment effects and reduce the reliability of the meta-analytic estimates. In conclusion, the present study suggests that acupoint application may be a potentially beneficial complementary therapy for patients with dysphagia after stroke, but the evidence is not conclusive.

A key issue regarding the outcomes is the use of “the effective rate,” which lacks international standardization. The definition and assessment criteria of effective rate may vary across the included studies, leading to inconsistencies in outcome measurement and limiting the clinical interpretability and generalizability of the results. This inconsistency may also contribute to heterogeneity among studies and affect the reliability of the combined results.

Traditional Chinese medicine believes that this disease is located in the brain, with symptoms in the throat, caused by phlegm and phlegm-blood congealing in the throat. Therefore, the treatment should mainly focus on resolving phlegm, opening the orifices, promoting blood circulation, and unblocking channels (19). Acupoint application refers to applying traditional Chinese medicine to acupoints, aiming to achieve the therapeutic purpose of unblocking meridians and harmonizing qi and blood through the dual effects of the pharmacological action of the medicine and the stimulation of the acupoints. The Tian Tu (CV22), Lian Quan (CV23), and Ren Ying (ST9) points are the main acupoints used in the treatment of post-stroke dysphagia. The combination of these three points can regulate the body's Qi and blood and unblock the meridians (24). Performing acupoint application therapy at these locations can enhance the coordination and flexibility of oral and pharyngeal muscles by stimulating the acupoints of the tongue and throat, thereby promoting the recovery of swallowing function (25).

This study utilized serum albumin levels and NRS2002 scores to reflect nutritional status. While the pooled results indicate that acupoint application combined with conventional rehabilitation training was superior to the conventional rehabilitation training alone in improving the nutritional status of patients with swallowing difficulties after stroke, the high risk of bias in the included studies urges cautious interpretation. The observed benefit might be explained by the fact that after acupoint application intervention, the swallowing ability of stroke patients is improved, allowing them to perform swallowing actions more smoothly, and the variety of foods patients could accept increased, along with greater food intake, which helps patients to obtain sufficient nutrition, thereby effectively improving the body's nutritional status. But it could also be exaggerated by methodological flaws such as lack of blinding.

Previous studies have shown that post-stroke dysphagia significantly impacts patients' quality of life (26), while early effective intervention can improve quality of life and reduce complications (27). The statistical results of this study suggest that acupoint application therapy is an effective complementary therapy for improving the quality of life in patients with post-stroke dysphagia. Possible reasons are as follows: (1) As patients' swallowing function improves, it can not only reduce the discomfort caused by choking and aspiration but also make eating smoother and increase nutritional intake, thus enhancing the patient's physical condition and reducing the negative impact of swallowing difficulties on daily life. (2) During acupoint application, the active ingredients of traditional Chinese medicine can improve the body's microcirculation by stimulating the acupoints, thereby promoting the survival and differentiation of hippocampal neurons, enhancing neuronal vitality, helping to restore the patient's limb function, and improving their mobility, thereby improving patients' quality of life (28). Again, the lack of rigorous methodological design in the included studies may lead to overestimation of the beneficial effect on quality of life, and these results need to be verified by more robust research.

This study still has some limitations: (1) The evidence is derived exclusively from small-sample, single-center RCTs conducted in China. This geographic homogeneity limits the external validity of the findings, making it uncertain whether they are generalizable to non-Chinese populations or applicable in broader healthcare settings where acupoint application is not routine. Confirmation through future multi-national research is needed; (2) None of the included trials reported allocation concealment or blinding of participants and outcome assessors, resulting in a high risk of bias; (3) Variations existed among included studies regarding treatment duration, acupoint selection, and composition of applied medications, which might impact on therapeutic efficacy; (4) The relevant studies lack data on long-term efficacy, safety, and follow-up results; (5) The funnel plot drawn based on treatment effectiveness shows incomplete symmetry. While this raises the possibility of publication bias, it could also indicate the presence of “small-study effects,” where smaller, methodologically weaker studies tend to report larger effect sizes, thereby potentially inflating the overall pooled estimate. Collectively, these limitations suggest that the positive findings may be fragile and susceptible to bias, further reducing our confidence in the pooled estimates and confirming that the overall certainty of the evidence is low.

Above limitations have important implications for how the findings should be interpreted clinically. While the statistically significant improvements across multiple outcomes—including swallowing function, nutritional status, and quality of life—point to clinically plausible benefits, the low certainty of evidence means these results should be viewed as hypothesis-generating rather than practice-changing. From a clinical perspective, the findings support considering acupoint application as an adjunctive therapy, but the evidence is only indicative and needs to be validated through more rigorous research. The observed benefits should inform future trial design rather than dictate current standards of care. Hence, future research should consider conducting large-scale, multicenter randomized controlled trials (RCTs) with adequate allocation concealment and blinding, in order to more comprehensively and objectively evaluate the efficacy of acupoint application for the treatment of post-stroke dysphagia.

## 5 Conclusion

In summary, acupoint application may be a potentially beneficial complementary therapy for dysphagia after stroke, not only improving the swallowing function of patients with post-stroke dysphagia but also enhancing their nutritional status and improving their quality of life. However, due to methodological limitations in the included studies (including high risk of bias, small sample sizes, and possible publication bias), the overall certainty of the evidence is low, and these results are suggestive rather than confirmatory. The description of acupoint application as an “effective complementary therapy” would overstate the strength of the current evidence. Therefore, the results should be interpreted with caution, and future research with large sample sizes, multiple centers, and rigorously designed methodologies is urgently needed to further validate the efficacy of acupoint application, so as to provide a reliable reference basis for clinical practice.

## Data Availability

The original contributions presented in the study are included in the article/supplementary material, further inquiries can be directed to the corresponding author.
